# Microstructural Effects in the Development of Near-Neutral pH Stress Corrosion Cracks in Pipelines

**DOI:** 10.3390/ma15134372

**Published:** 2022-06-21

**Authors:** Ci Zhang, Minrui Ran, Yao Wang, Wenyue Zheng

**Affiliations:** 1 National Center for Materials Service Safety, University of Science and Technology Beijing, Beijing 100083, China; zhangxiaoci1208@163.com (C.Z.); 18600179244@163.com (M.R.); 2 South China Branch of National Petroleum and Natural Gas Pipe Network Group, Guangzhou 510620, China

**Keywords:** pipeline steel, stress corrosion cracking, interrupted SSRT, corrosion, stress threshold, near-neutral pH

## Abstract

The corrosion and stress corrosion cracking (SCC) behaviors of 20#, X60, and X80 pipeline steels in a near-neutral pH environment were investigated by means of electrochemical measurement, immersion test, and interrupted slow strain rate tensile (SSRT) test. The propensity for SCC, as indicated by the stress threshold value for crack initiation, was found to be dependent on the type of steel microstructure. Cracks were initiated in the high-strength steel X80 at a stress less than its yield strength, whereas in the other lower-grade steels, the initiation of cracks occurred after the yielding point. The threshold stress of SCC initiation in the near-neutral pH environment for 20#, X60, and X80 steels were 130.64% σ_ys_, 106.79% σ_ys_, and 86.92% σ_ys_, respectively. The SCC of 20# and X60 were characterized by the formation of transgranular and intergranular cracks, while X80 steel was only by transgranular cracking. The occurrence of corrosion had a great effect on crack initiation and the growth at the later stage. The latter involved hydrogen effects. A correlation between SCC sensitivity and the yield strength of the steel has been identified.

## 1. Introduction

Since near-neutral pH SCC was first discovered in Canadian gas transmission pipelines in the mid-1980s [1], many pipeline leakages and ruptures were ascribed to SCC. This type of cracking develops on the external surface of the pipeline where the trapped water under the disbonded pipeline coating is a dilute electrolyte with a pH in the range of 6.5 to 8.0 [1]. This form of environmentally assisted cracking has since been reproduced in a wide range of steel grades. The 20# and X60 steels are examples of older in-service pipelines. With the increasing demand for pipeline transport, X80, as a typical high-grade pipeline steel, is now being widely used, especially in Asia. The associated microstructure types have evolved from ferrite-pearlite for steels of strength lower than X60 to acicular-ferrite/bainite for X80 and higher-grade steels.

The near-neutral pH SCC cracks are usually characterized by transgranular cracking with a certain amount of corrosion at the crack mouth, indicating that some corrosion is taking place concurrently on the crack walls as the crack front deepened [2]. In general, the influencing factors of SCC mainly include the soil environment, the nature of the structure, and the loading condition. The most probable mechanism of near-neutral pH SCC is a complex one that involves, to some extent, some type of hydrogen-induced embrittlement, as proposed by Parkins [3] and others. As the formation of a corrosion product layer on stressed steel does not provide complete protection to the underlying metal due to the loose and porous structure of the surface film, the rate of dissolution of steel can increase significantly with the application of stress, particularly when the stress level is increased to 80% of the yielding strength of steel [4]. In return, localized corrosion and pitting can cause stress concentration, which often becomes the initiation site of SCC [5].

Although the role of dissolution in the crack initiation process seems essential, particularly when the steel is under free-corrosion conditions, further penetration of the crack is not solely by dissolution. For example, Parkins [6] and Harle [7] found that the SCC crack growth predicted using Faraday’s law is far less than the actual observed growth rate if the existing anodic dissolution model is used; thus, a certain type of mechanical contribution involving hydrogen must be provoked to explain the actual processes of near-neutral pH SCC.

As one of the three key factors affecting the SCC of pipelines, materials often cause differences in sensitivity through uneven microstructure and defects [1,6]. The microstructure types of high-strength steel (mainly ferrite, bainite, and M/A) and low-strength steel (ferrite and pearlite) are obviously different. The number of dislocations and inclusions produced by different manufacturing processes and alloying degrees is also significantly different. The near-neutral pH SCC cracks are usually characterized by transgranular cracking with a certain amount of corrosion at the crack mouth, indicating that corrosion was taking place concurrently on the crack walls as the crack front deepened [2]. Investigation of the effect of different microstructural types on anodic dissolution helps understand the SCC mechanism [5]. There is a report that the hard and brittle phases in steels are more prone to SCC crack propagation [8]. For the newer steel grades such as X70 and X80, the combination of acicular ferrite and bainite may be the optimum combination of strength and ductility [9]. However, there is no well-established relationship between the microstructure of the pipeline steel and its stress corrosion sensitivity.

In this study, the microstructural effects on corrosion and SCC sensitivity were studied using 20#, X60, and X80 pipeline steels. Electrochemical and immersion tests evaluated the different corrosion tendencies in a soil-water simulation solution called NS4 and the SCC sensitivity was tested using the interrupted SSRT technique. After the SSRT test, the SCC sensitivity of different type steels was evaluated by measuring the threshold stress for crack initiation in each type of sample. Together with SEM and optical observations of the crack morphology, we attempted to produce an in-depth understanding of the cracking process and the roles of microstructure in the development of SCC in a near-neutral pH environment.

## 2. Materials and Test Methods

### 2.1. Materials

The 20#, X60, and X80 samples were taken from stock pipeline segments in a commercial pipeline company, with outside diameters of 426.00 mm, 1219.00 mm, and 455.00 mm, and the corresponding wall thickness is 45.00 mm, 26.00 mm, and 11.00 mm, respectively. All the test samples in this work were taken in the middle of the pipe wall thickness, with the sample length perpendicular to the rolling direction. Three parallel samples were tested for each steel. Table 1 list the chemical compositions of 20#, X60, and X80 line pipe steels. The S and P contents for three pipeline steels are controlled at a low level (Table 1). The carbon concentration of X60 steel is the highest (0.115 wt. %) among the three steels, while the content of other alloying elements is the lowest in this material. The content of Mo is significantly higher in steel 20# than in X60 and X80. The 20# and X60 steels were processed by hot rolling, whereas the X80 were obtained by thermomechanical controlled processing (TMCP).

### 2.2. Microstructural Characterization

For microstructural observations, all tested specimens were ground to 2000 grit paper and polished with 2.5 μm diamond suspension. They were degreased with alcohol and etched with nital solution. The microstructure was examined with an optical microscope (OM, DM4000M from Wetzlar, Germany) and scanning electron microscope (SEM, SIGMA 300 from Germany).

### 2.3. Electrochemical Measurement

The electrochemical experiments, including open-circuit potential, and potentiodynamic polarization measurements, were performed in a three-electrode cell using a P4000 workstation (Gamry from Warminster, PA, USA). A saturated calomel electrode (SCE from Shanghai, China) was used as the reference electrode, and a platinum sheet was used as the counter electrode. The 20#, X60, and X80 pipeline steels with an area of 1 cm^2^ were used as the working electrode. The electrode surface was polished with sandpaper up to 1500 grit, and then by diamond paste up to 0.25 μm, followed by cleaning with deionized water and alcohol. Parallel samples were arranged for each test, and the average values of the three test results were taken. After the cell was set up, the working electrode was first stabilized for about half an hour at open-circuit potential before direct current (DC) polarization started. The polarization curves were measured at a potential sweeping rate of 0.33 mV/s in a potential range of −0.6 V_SCE_ to 0.0 V_SCE_ (the electrochemical potential value, in volt, relative to a saturated calomel electrode, SCE). All electrochemical data was analyzed using Gamry Echem Analyst software (version number 6.04, Warminster, PA, USA).

All test solutions in this study were NS4 solutions, a near-neutral pH solution, simulating the electrolyte trapped under disbanded coating in Canada [6,9,10,11]. The chemical composition of NS4 solution was as follows (g/L): KCl 0.122, NaHCO_3_ 0.483, CaCl_2_·2H_2_O 0.181, and MgSO_4_·7H_2_O 0.131. The solution was made using distilled water and analytical-grade chemical reagents. To achieve an anaerobic and near-neutral pH condition with a pH value of about 6.4, the solution was purged with N_2_ gas for half an hour before each test, and then the mixed gas of 5% CO_2_ and 95% N_2_ throughout the experiment. All tests were performed at ambient temperature.

### 2.4. Immersion Test

Samples were cut into 50 × 10 × 3 mm^3^ for the immersion test. All samples were polished with sandpaper up to 1500 grit and cleaned with deionized water and alcohol. Each sample was weighed and immersed in the test solution for 14 days in a glass tank with a screw cap. After the experiment, the corrosion products formed on the specimen were removed by descaling liquid (500 mL concentrated hydrochloric acid mixed with 500 mL deionized water, inhibited with 10 g hexamethylenetetramine), and then cleaned by the ultrasound with pure ethanol for 15 min, dried, and finally weighed.

The corrosion rate was calculated by the following ASTM G1-03 standard [12]:

(1) $$\mathrm{Corrosion}\;\mathrm{rate}=\frac{K\;\times \;w}{\rho \mathrm{AT}}$$

where, K is a conversion coefficient (3.54 × 10^6^ mils per year (mpy)), and w, ρ, A, and T are the total weight loss (g), the density (g/cm^3^), the area of the specimen (cm^2^), and the test time (h), respectively.

### 2.5. Interrupted Slow Strain Rate Tensile (SSRT) Test after Immersion

**Figure 1:**
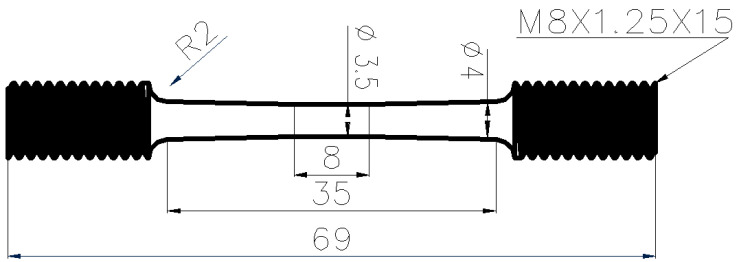
Dimension of test specimen (unit: mm).

All samples were machined into a dog-bone tapered shape for the SSRT tests, with their length direction perpendicular to the rolling direction of the materials. In order to obtain the threshold value of SCC initiation, the normal dog-bone specimen was modified to include a tapered section along the length of specimen, with 35.00 mm total working section length, 8.00 mm in the central gauge length, and 3.50 mm gauge diameter in the center, while the diameter at the end of the tapered section (the shoulder) is 4.00 mm, as shown in Figure 1. This way, a stress gradient was produced on the tapered specimen, and the threshold stress value from a single test can be determined by observing the exact initiation position along the gauge length, as the actual stress level decreased from the center of the sample to its shoulders where the diameter is increased. Before each test, the tensile specimens were ground sequentially from coarse to fine grades to finally 1500 grit emery papers, with the final polishing direction parallel to the applied stress.

The interrupted SSRT experiment was carried out in two steps through a Letry20007-L25 test system. The first step of the interrupted SSRT tests was performed at a strain rate of 1 × 10^−6^/s, at which the stress reached the ultimate tensile strength (UTS) of the steel. During the second step of the experiment, the steel samples were held at a constant load equaling to the UTS level while the samples stayed immersed in NS4 solution for 24 h, 48 h, and 72 h, respectively. The tensile strengths of 20#, X60, and X80 steels are ~481.00 MPa, ~553.00 MPa, and ~701.00 MPa, respectively. The final elongation at break of the three samples is ~24.93%, ~22.75%, and ~22.80%, respectively.

## 3. Results

### 3.1. Microstructural Characterization

**Figure 2:**
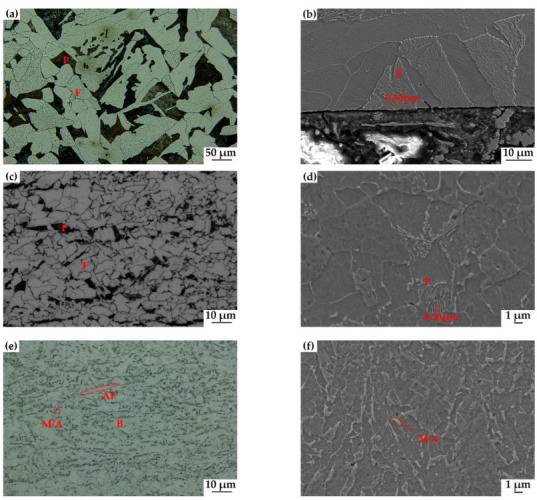
Microstructures of 20# (**a**,**b**), X60 (**c**,**d**), and X80 (**e**,**f**) steel. (P: Pearlite; F: Ferrite; B: Bainite; AF: Acicular Ferrite; M/A: Martensite/Austenite).

Both 20# and X60 steels contained some micro-alloy elements and were produced by the hot rolling process, resulting in similar types of structural compositions. The optical micrographs of the nital etched steel specimens are shown in Figure 2. The pearlite (P) and massive ferrite (F) are evident for the 20# and X60 steels. The volume percentages of the pearlite and ferrite in 20# steel are estimated to be 29% and 71%, respectively, by statistical image analysis. The grain size is about 26 μm and the pearlite lamellar spacing is about 0.65 μm, as shown in Figure 2a,b. The volume percentages of the pearlite and ferrite in X60 steel are estimated to be 12% and 88%, respectively. The grain size is about 8 μm, and the pearlite lamellar spacing is about 0.23 μm, as shown in Figure 2c,d. X80 steel was processed by combining a high degree of micro-alloying and a thermomechanical controlled process (TMCP). Figure 2e,f shows that X80 steel consists of acicular ferrite (AF)/granular bainite (GB) with some islands of M/A.

### 3.2. Corrosion Rates of 20#, X60, and X80 Pipeline Steels

#### 3.2.1. Electrochemical Measurements of 20#, X60, and X80 Pipeline Steels

**Figure 3:**
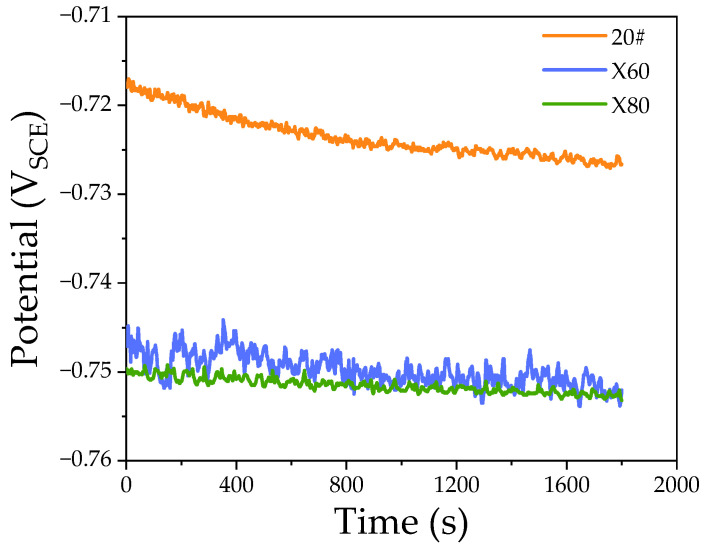
Open-circuit potential and test duration of 20#, X60, and X80 steels in NS4 solution.

Figure 3 shows the evolution of open-circuit potential (OCP) with time in the NS4 solution. The corrosion potential of 20# steel was −718 mV_SCE_, significantly higher than that of X60 and X80 steels. The corrosion potentials of X60 and X80 steels were −747 mV_SCE_ and −750 mV_SCE_, respectively, which were similar to each other.

**Figure 4:**
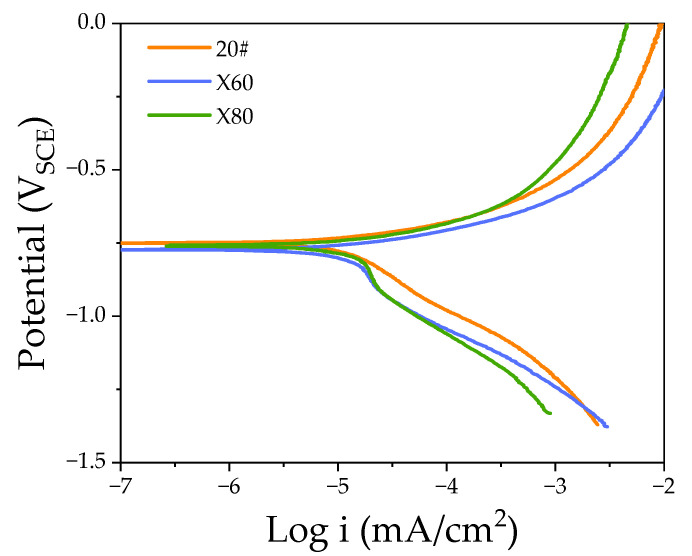
Polarization curve of 20#, X60, and X80 steels in NS4 solution.

**Figure 5:**
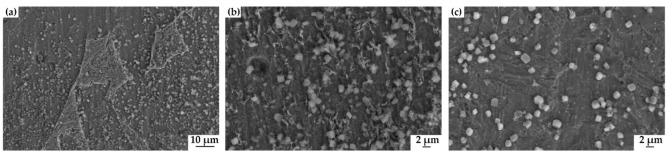
The SEM micrograph of 20# (**a**), X60 (**b**), and X80 (**c**) steels after the polarization test.

The polarization curve of 20#, X60, and X80 steels in NS4 solution, as shown in Figure 4, is analyzed employing iterative curve-fitting, and the analysis results are shown in Table 2. E_corr_ is the corrosion potential of corrosion systems (mV_SCE_), i_corr_ is the corrosion current density (μA/cm^2^), and Beta A and Beta C are the anodic and cathodic Tafel slopes, respectively. C_r_ is the corrosion rate of the sample. i_corr_ of the three steels is 6.48 µA/cm^2^, 13.47 µA/cm^2^, and 14.67 µA/cm^2^, respectively, and the corresponding corrosion rates C_r_ of the three samples are 3.01 mpy, 6.25 mpy, and 6.80 mpy, respectively. Figure 5 is the SEM micrograph of the test sample after the polarization test.

#### 3.2.2. Weight Loss Measurements of 20#, X60, and X80 Steels

**Figure 6:**
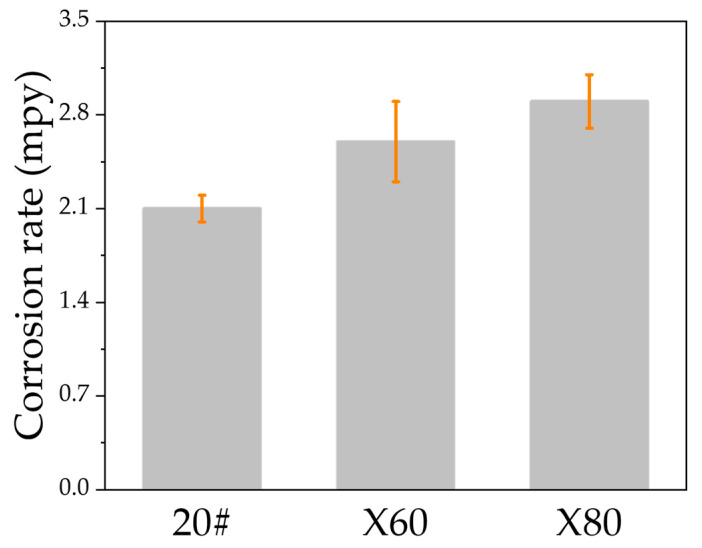
Corrosion rates of 20#, X60, and X80 steels determined from the weight-loss test after 14 days immersion.

**Figure 7:**
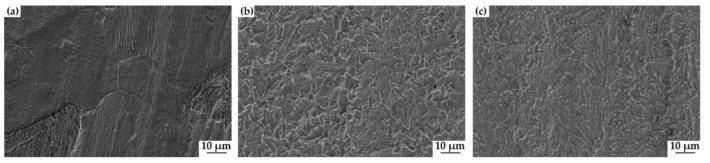
The morphology of 20# (**a**), X60 (**b**), and X80 (**c**) after removal of corrosion products after 14 days immersion.

Figure 6 shows the corrosion rates of the steels 20#, X60, and X80, determined from the weight loss after 14 days of testing in NS4 solution, which is calculated by Equation (1). The average corrosion rate of 20# steel (2.10 mpy) is the smallest, and X80 steel has the highest average corrosion rate (2.90 mpy). The corrosion rate of samples of X60 steel is 2.60 mpy. Figure 7 shows the morphology of the samples after the removal of the corrosion products at the completion of the weight loss tests.

### 3.3. SCC Susceptibility of Steels 20#, X60, and X80 Steels

#### 3.3.1. Initiation Site of SCC

**Figure 8:**
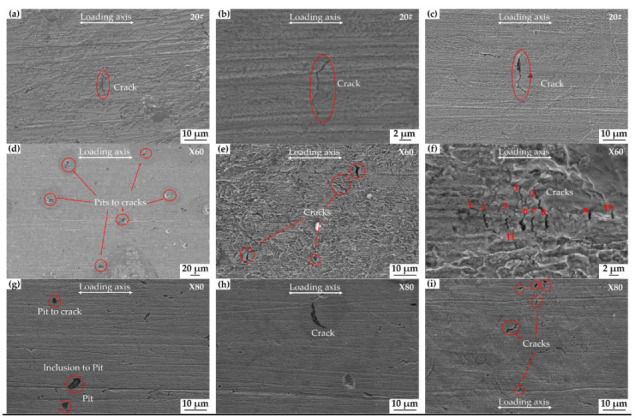
Surface morphology of 20# (**a**–**c**), X60 (**d**–**f**), and X80 (**g**–**i**) after SSRT to UTS level and immersion for different times in near-neutral pH environment: 24 h: (**a**,**d**,**g**), 48 h: (**b**,**e**,**h**), 72 h: (**c**,**f**,**i**).

All three steels surfaces exhibit varying degrees of surface deterioration due to corrosion exposure. The extent of corrosion and the cracking characteristics (the number, depth, and length of cracks) become more and more evident with the increase in the immersion time, as shown in Figure 8. Compared with X60 and X80, the corrosion of 20# steel is the lightest, while the crack developed is the longest. Figure 8a–c shows the surface morphology of 20# steel after the SSRT test as follows: after the applied stress had reached the UTS point, the samples were held at load for 24, 48, and 72 h, respectively in a near-neutral pH solution. It is clear that the surface length cracks on the 20# steel become longer with the increase in test time.

Figure 8d–f shows the surface morphology of X60 steel after holding the samples at UTS level for 24, 48, and 72 h, respectively. After 24 h, there are many barely visible microcracks, often at the sites of pits (Figure 8d). As the immersion time is extended to 48 h, a clearer morphology of the cracks appears on the surface, Figure 8e. After 72 h of immersion, intensive cracking is seen, as in Figure 8f.

X80 steel shows a trend similar to X60 steel. Figure 8g–i shows that the extent of surface corrosion increases with the immersion time. Some pits formed at the inclusion sites and cracks from the pit position are shown in Figure 8g. Many cracks appeared on the surface after 72 h of immersion, as shown in Figure 8i. These different crack density numbers, lengths, and morphologies reflect the differing sensitivity of different pipeline steels.

#### 3.3.2. The Threshold Stress for SCC Initiation

The threshold stress (σ_th_) of SCC initiation was calculated by measuring the position where the cracking vanishes on the tapered sample. From the corresponding cross-section area at this point and the total load value, the stress at the locations where cracks were no longer seen can be calculated as follows:

(2) $$\sigma_{\mathrm{th}}=\sigma_{\max}\;\cdot \;f_{\mathrm{location}}$$

(3) $$f_{\mathrm{location}}=\frac{\mathrm{cross}\;\sec\mathrm{tion}\;\mathrm{area}\;\mathrm{in}\;\mathrm{the}\;\mathrm{middle}\;\mathrm{of}\;\mathrm{gauge}\;\mathrm{length}}{\mathrm{cross}\;\sec\mathrm{tion}\;\mathrm{area}\;\mathrm{of}\;\mathrm{SCC}\;\mathrm{initiation}\;\mathrm{point}}$$

where σ_th_ is the threshold stress for SCC initiation, σ_max_ is the applied stress at the middle of the gauge length, and f_location_ is the location factor.

**Figure 9:**
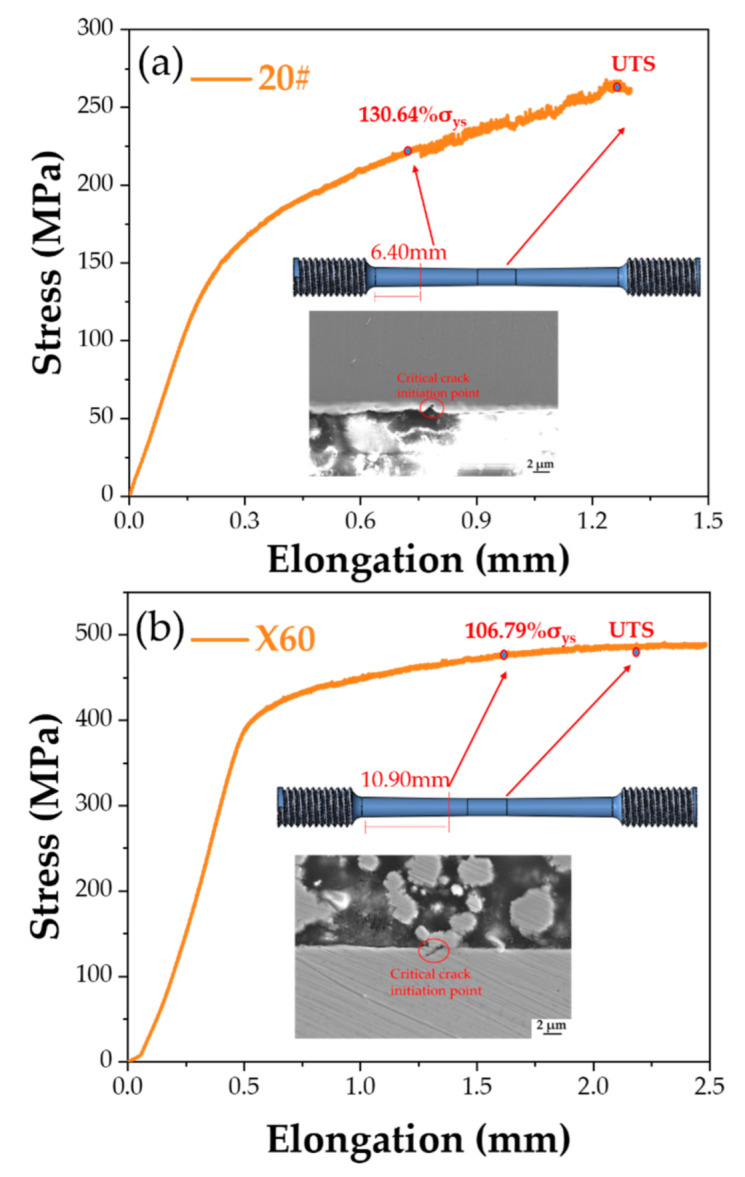

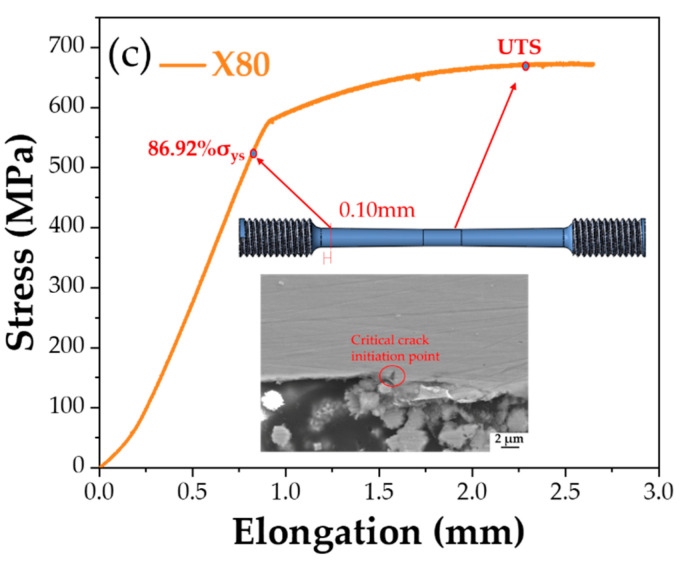
Determination of threshold stress for crack initiation (**a**) 20#, (**b**) X60, and (**c**) X80.

Figure 9 shows the points for crack initiation of 20#, X60, and X80 steels on their stress-total displacement curves, in which the stress is the total load divided by the cross-section area at the mid-length of the sample and total displacement is the distance traveled by the crosshead of the test machine. The calculation process of the threshold stress of SCC for 20#, X60, and X80 steels is listed in Table 3, The threshold stress for SCC to form corresponded to 130.64% σ_ys_ for 20# steel, 106.79% σ_ys_ for X60 steel, and 86.92% σ_ys_ for X80 steel, respectively.

#### 3.3.3. The Cross-Sectional Morphology of SCC Cracks

**Figure 10:**
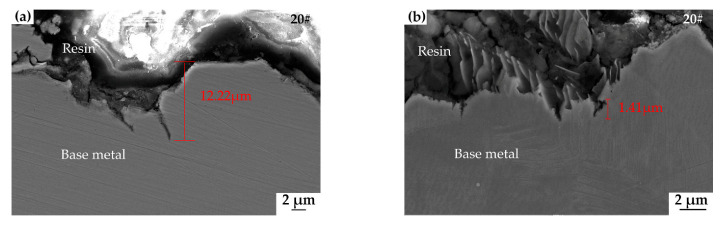

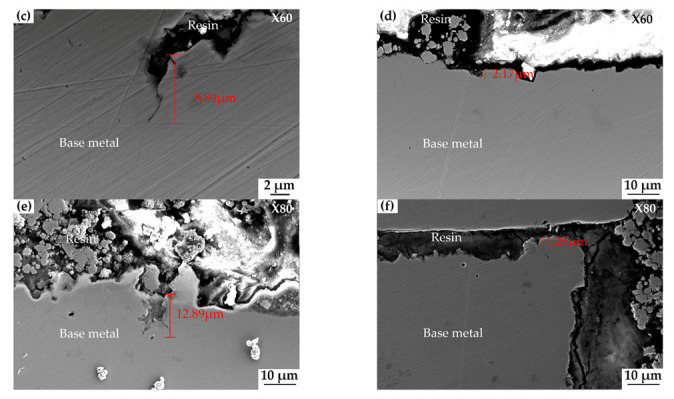
The crack growth morphology at different positions on the cross-section 20#, X60 and X80 pipeline steels after SSRT to the UTS level for 48 h: middle section: (**a**,**c**,**e**), lateral section: (**b**,**d**,**f**).

Figure 10 shows the crack cross-sectional morphology at the central and shoulder locations of the 20#, X60, and X80 pipeline steels after SSRT to the UTS level for 48 h. The SCC initiation mode and depth can be obtained from these images. Figure 10a,c,e show the characteristics of crack penetration in the middle section, which is also the maximum stress point for 20#, X60, and X80 pipeline samples. While many cracks in all three steels are initiated at the bottom of the pits, there are some cracks initiated on the flat surface. As described earlier, the stress of the central section of the tapered sample is the highest, descending in a gradient along the sample shoulder. As a result, the crack will disappear on the shoulder when the stress is below the threshold value for crack initiation.

From the stress-strain measurements of the 20#, X60, and X80 steels, the time interval for the load to pass the threshold level and the final maximum UTS point was calculated as 2.57 d, 4.32 d, and 2.84 d, including the 2-day constant-load holding time, respectively, as shown in Table 4. Figure 10 shows the crack depth difference between the middle section and the threshold stress point. The maximum crack growth rate can be estimated by knowing the maximum crack depth and time interval. Figure 10a,c,e show that the crack depth of the 20#, X60, and X80 steels in the middle section is 12.22 μm, 8.89 μm, and 12.89 μm, respectively. Figure 10b,d,f show the cracks appearing at the threshold stress point of the 20#, X60, and X80 steels, respectively. The maximum crack growth rate of 20#, X60, and X80 steels was calculated to be 4.75 μm/d, 2.06 μm/d, and 4.54 μm/d, respectively; these are shown in Table 4. It should be noted that this maximum crack growth rate is the average growth rate of the deepest crack.

**Figure 11:**
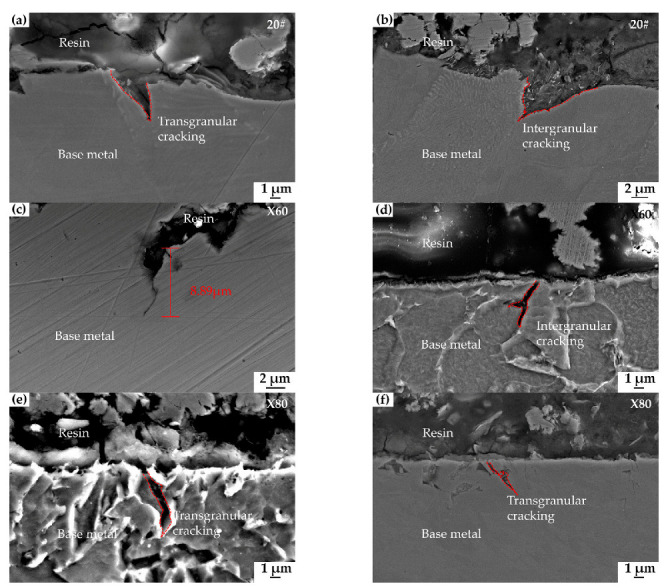
Early crack propagation morphology of different steels after SSRT to the UTS level and immersion for 48 h in a near-neutral pH environment: 20# (**a**,**b**), X60 (**c**,**d**), and X80 (**e**,**f**).

Figure 11 shows the early crack propagation paths of different steels after slow-straining to the UTS level and immersion for 48 h in a near-neutral pH environment. The direction in which the crack propagates is generally vertical to the applied axial stress. Most cracks are initiated by corrosion sites and gradually develop into sharp cracks. Figure 11a,b shows that the SCC cracks of 20# steel originate at the bottom of extensive corrosion pits. The crack propagates inside a pearlite grain in Figure 11a; it can also develop along with the interface between the pearlite and ferrite, as in Figure 9b. Figure 11c,d show the SCC cracks of X60 steel. A crack is seen to propagate through pearlite in Figure 11c and the cracking is at the interface between ferrites in Figure 11d; this aspect is similar to 20# steel. Figure 11e,f shows that the SCC cracks of X80 steel originate and propagate transgranularly through the ferrite/granular bainite.

## 4. Discussion

### 4.1. SCC Mechanism of 20#, X60, and X80 Steels in a Near-Neutral pH Environment

The following electrochemical reactions occur when pipeline steels are put in an NS4 solution purged with 5% CO_2_ and 95% N_2_ mix gas. There are the dissolution of iron and the formation of a layer of iron hydroxide deposit are as follows [13,14]:

(4) $${\mathrm{Fe}}^{2+}+{\mathrm{OH}}^{-}\to \mathrm{Fe}{\left(\mathrm{OH}\right)}_{2}+2e^{-}$$

(5) $$\mathrm{Fe}+2H_{2}O\to \mathrm{Fe}{\left(\mathrm{OH}\right)}_{2}+2H^{+}+2e^{-}$$

In anaerobic conditions, the reduction of bicarbonate, water, and hydrogen ions are involved in the cathodic reactions as follows [15,16]:

(6) $$H_{2}O+e^{-}\to H\left(\mathrm{ads}\right)+{\mathrm{OH}}^{-}$$

(7) $$H_{2}{\mathrm{CO}}_{3}+e^{-}\to H\left(\mathrm{ads}\right)+{\mathrm{HCO}}_{3}^{-}$$

(8) $${\mathrm{HCO}}_{3}^{-}+e^{-}\to H\left(\mathrm{ads}\right)+{\mathrm{CO}}_{3}^{2-}$$

(9) $$H^{+}+e^{-}\to H\left(\mathrm{ads}\right)$$

The corrosion product FeCO_3_ is produced by electrochemical or chemical reactions [17].

(10) $$\mathrm{Fe}+{\mathrm{HCO}}_{3}^{-}\to {\mathrm{FeCO}}_{3}+H^{+}+2e^{-}\;(\mathrm{electrochemical})$$

(11) $${\mathrm{Fe}}^{2+}+{\mathrm{HCO}}_{3}^{-}\to {\mathrm{FeCO}}_{3}+H^{+}\;(\mathrm{chemical})$$

The morphology of the corroded surface of steels after immersion has been presented in Figure 8. Under tensile loading, SCC cracks on the surface of 20#, X60, and X80 steels formed, and these cracks are seen in Figure 10 and Figure 11. We can see from the comparison of the general corrosion rate and the maximum crack penetration rate that the anodic dissolution alone is not enough to account for the crack depth achieved in this near-neutral pH SCC process. The weight-loss test is a direct way to estimate the possible contribution of corrosion to the growth of cracks [10]. If the crack penetration had occurred by a pure dissolution process, we could estimate the rate of the cracking using the Faraday law and the dissolution rate. In the present study, using the rate of corrosion reported in Section 3.2.2, the total depth of 20# steel cracks could reach only 0.43 µm under the experiment time. Similarly, X60 and X80 steels can only develop cracks that are 0.53 µm and 0.57 μm deep, respectively. As shown in Figure 10, the maximum crack depths of 20#, X60, and X80 steels are, in fact, 12.22 μm, 8.89 μm, and 12.89 μm, respectively, which is much more than the value predicted using the Faraday law. In fact, all the crack walls near the crack mouth showed corrosion up to a fraction of a micrometer, and this is consistent with the general corrosion rate from the weight loss test. In the propagation direction, the depth of penetration is many times the corrosion amount of the crack wall. Therefore, a simple anodic dissolution model cannot explain the SCC propagation mechanism of these steels in a near-neutral pH environment.

Many studies have shown that both stress [18,19] and local hydrogen [18,19,20] promote the anodic dissolution of iron, and a synergistic effect is likely involved in producing the crack growth that we have seen. The different microstructures of the material rendered the difference in corrosion rate, and stress concentration due to pitting and inhomogeneous microstructure further aggravates crack formation by accelerating corrosion and increasing the local stress [21].

The dissolution of the crack tip generates many metal cations (Fe^2+^) [13]. The cations are hydrolyzed at the crack tip, resulting in the acidification of the crack tip. The difference in pH value between the crack tip and the matrix environment can also accelerate the dissolution of the crack tip. In addition, the H atoms generated by excessive H^+^ can be reduced to atomic H and can be attracted to the stress concentration area, gathering in the crack tip region, resulting in a hydrogen-embrittlement type of cracking. J. Been et al. [22] measured the hydrogen concentration of polished and mill scaled X65 by a hydrogen permeation test of 0.73 mol/m^3^ and 0.38 mol/m^3^, respectively. Moreover, the hydrogen concentration at the crack tip position would be more elevated due to the strain concentration in front of the crack tip, which was also reported in the literature [23]. The aspects of hydrogen embrittlement in the cracking process have been studied by others [22,24]. Therefore, the crack extension of 20#, X60, and X80 steels in a near-neutral pH environment can be a result of the combined action of anodic dissolution and hydrogen-induced embrittlement.

### 4.2. Effect of Microstructure on SCC Sensitivity

Different steel compositions combined with processing techniques lead to noticeable microstructure differences among the three pipeline steels. The electrochemical parameters of 20#, X60, and X80 steels reflected the noticeable differences in the corrosion rates in a near-neutral pH environment. The anodic polarization curves show active corrosion behavior of 20#, X60, and X80 steels in a near-neutral pH environment and suggest a poor protective ability of the corrosion product layer. The small decline of X60 and X80 in the cathodic polarization section, around −1.0 V of the polarization curve, indicates the formation of cathodic deposits [14]. The E_corr_ and i_corr_ in Table 2 show that the 20# sample is electrochemically less active than X60 and X80 steels in a near-neutral pH environment. The thermodynamic stability of materials decreases when the corrosion potential becomes more negative. It is meant that the electrochemical stability of 20# steel is superior to that of X60 and X80 steels. Alloying elements are effective factors affecting corrosion resistance [25,26]. The content of Mo in 20# steel is significantly higher than those in X60 and X80 steel, as shown in Table 1, which may be the main reason for its superior corrosion resistance. As a micro-alloyed high-strength steel, the total elemental content in X80 steel is higher than in 20# and X60 steels, and its corrosion tendency is more pronounced [27].

From Figure 8, it can be seen that the SCC cracks can be initiated from the surface of the sample where there are pits. The inclusions or M/A microstructure in the X80 steel can serve as pitting sites. These inclusions in our tested steels are those enriched with Si, Al oxide, ferric carbide, Si–ferric carbide, and Al-Ca-O mixture. The most common inclusions are Si and Al oxides [28,29]. Studies have shown that the M/A structure is more susceptible to corrosion than pearlite and bainite [27]. The stress concentration and the difference in corrosion tendency between these inclusions, or M/A and matrix, result in the initiation of cracks or pitting, which could later propagate and penetrate into the steel.

The SSRT test is a common method to evaluate the SCC sensitivity. Many studies [30,31,32] qualitatively compare the SCC sensitivity between materials by measuring the section shrinkage and elongation of samples after a sample reaches its fracture point. In the present study, by using a tapered sample and by straining the test sample only to the UTS point of the thinnest section of the sample, the threshold stress value of the SCC samples can also be obtained to evaluate the SCC resistance [33]. The threshold stress for SCC crack to appear of 20#, X60, and X80 steels is 130.64% σ_ys_, 106.79% σ_ys_, and 86.92% σ_ys_, respectively. The higher the pipeline strength, the lower the threshold stress (the ratio of the SCC threshold to the yield strength) of the material. The SCC cracking in X80 steel happened at the lowest percentage yield strength level among the three. With the increase in material strength, its SCC sensitivity increases, which is consistent with the findings of related researchers [34,35] that use other techniques for SCC evaluation. Lu, B.T [35] defines the ratio of tensile pipeline steels reduction in area in the test solution to that in the air as the SCC resistance of the sample. Their results show the SCC resistance of similar type microstructure decrease with increasing yield strength.

Different microstructure affects the SCC sensitivity of steel and its crack propagation path. The microcracks in Figure 10 and Figure 11 are generally wide and transgranular, with most cracks propagating through the grains of pearlite and a few others extending along with the interface of pearlite and ferrite. 20# steel has good corrosion resistance due to its higher Mo content, and therefore, less anodic dissolution is involved in the SCC cracking process of 20# steel than in X60 and X80 steels. Besides, the significant difference in the hardness of ferrite and lamellar pearlite produces a significant stress concentration effect at interfacial boundaries [36]. As a result, 20# steel has grain-penetrating cracks inside the pearlite and a lot of intergranular cracks along the boundary between pearlite and ferrite.

X60 is a controlled-rolled micro-alloyed steel. The microstructure of X60 is ferrite and pearlite, similar to 20# steel. Because of the significant difference in hardness between the two phases, which exhibit transgranular and intergranular cracks in a near-neutral pH environment. This mixed cracking mode of low-strength steel cracking also appeared in the study of Chu, R. et al. [37]. The different proportions of pearlite and ferrite caused different degrees of transgranular cracks in 20# and X60 steel. The microstructure of X60 steel with a smaller amount of pearlite is more uniform than that of steel 20#, resulting in mostly transgranular SCC cracking. It seems that the difference in lamellar spacing of pearlite leads to different resistances to crack propagation as follows: the crack propagation rate of 20# steel, with a bigger pearlite lamellar spacing, is significantly higher than the X60.

As a high-strength linepipe steel, X80 is considered for new pipeline construction because of its higher efficiency for gas transportation. To achieve high strength and high fracture toughness, there are more alloying elements added to X80 steel. However, SCC sensitivity was not considered in designing steel chemistry. As there are more precipitates and secondary phases such as M/A islands in the X80 steel, inhomogeneities in the microstructure favor uneven local stress distribution, and, subsequently, there are more stress concentration sites in the X80 steel than in the other two steels. Inclusions, such as hydrogen traps, can increase the content of hydrogen in the steel microstructure. In addition, pitting also boosts the local concentration of hydrogen, which can also significantly increase the SCC sensitivity of the material. In our study, X80 exhibits the lowest threshold stress of all three steels for SCC, as shown in Figure 9 and Table 3. Therefore, robust anti-corrosion coatings for the X80 pipeline are essential.

## 5. Conclusions

The microstructural effects on the SCC sensitivity of three steels in a near-neutral pH environment were evaluated. The conclusions are as follows:

(1) The corrosion rates of 20#, X60, and X80 steels were 2.1 mpy, 2.6 mpy, and 2.9 mpy, respectively, in the near-neutral pH environment. The highest-strength X80 steel shows the highest corrosion rate (2.9 mpy);
(2) Although the occurrence of corrosion had a significant effect on crack initiation in creating stress-concentrating sites such as pits and in acidifying the pH within pits, the anodic dissolution rate is not enough to account for the crack depths of SCC of 20#, X60, and X80 steels;
(3) The SCC of 20# and X60 were characterized by transgranular and intergranular crack, while X80 steel by transgranular crack;
(4) Using a tapered sample and straining the test sample only to the UTS point of the sample, the threshold stress value for SCC initiation can also be obtained to evaluate the SCC sensitivity. Cracks were initiated in the high-strength steel X80 at a stress level less than its yield strength, whereas in the other lower-grade steels the initiation of cracks occurred after the yielding point. The threshold stress of SCC initiation in the near-neutral pH environment for 20#, X60, and X80 steels were 130.64% σ_ys_, 106.79% σ_ys_, and 86.92% σ_ys_, respectively.

## Figures and Tables

**Table 1:** Chemical composition of 20#, X60, and X80 steels (wt. %).

Material	Mn	Si	P	Mo	C	S	Ni	Cr	Cu	Nb	Ti	Al	V
20#	1.71	0.218	0.009	0.23	0.057	0.001	0.016	0.228	—	—	—	—	0.025
X60	1.28	0.276	0.008	0.002	0.115	<0.001	<0.001	<0.001	0.017	0.018	0.014	0.026	—
X80	1.70	0.207	0.01	<0.001	0.056	0.002	0.085	0.22	0.194	0.046	0.016	0.033	—

**Table 2:** Electrochemical parameters of 20#, X60, and X80 steels in NS4 solution.

Parameters	20#	X60	X80
E_corr_ (mV_SCE_)	−719.00	−772.00	−757.50
i_corr_ (mA/cm^2^)	6.48 ± 0.12	13.47 ± 0.15	14.67 ± 0.14
Beta A (V·dec^−1^)	0.05 ± 0.02	0.07 ± 0.01	0.08 ± 0.01
Beta C (V·dec^−1^)	0.19 ± 0.01	0.20 ± 0.02	0.22 ± 0.02
C_r_ (mpy)	3.01 ± 0.12	6.25 ± 0.15	6.80 ± 0.14

**Table 3:** Threshold stress for SCC initiation of 20#, X60, and X80 steels.

Material	Yield Stress (MPa)	Stress for SCC to Appear (MPa)	Ratio of Threshold Stress/Yield Stress (%)
20#	174.59	228.08	130.64
X60	414.00	442.11	106.79
X80	581.47	505.40	86.92

**Table 4:** The average SCC growth rate of the deepest crack in the 20#, X60, and X80 steels.

Material	Time for Crack Growth (d)	Max Crack Depth (µm)	V = Depth/t (µm/d)
20#	2.57	12.22	4.75
X60	4.32	8.89	2.06
X80	2.84	12.89	4.54

## Data Availability

Date are available on request to the corresponding author.
